# Microplastics, Nanoplastics and Heart Contamination: The Hidden Threat

**DOI:** 10.3390/jcm14217618

**Published:** 2025-10-27

**Authors:** Gian Luca Iannuzzi, Michele D’Alto, Giorgio Bosso, Antonio Pio Montella, Veronica D’Oria, Luigi Pellegrino, Giuseppe Boccaforno, Alessandro Masi, Antonio Orlando, Renato Franco, Andrea Ronchi, Carmine Nicastro, Marisa De Feo

**Affiliations:** 1 Local Health Autorithy, Service of Cardiology, 82100 Benevento, Italy; 2 Department of Cardiology, Monaldi Hospital, 80131 Naples, Italy; mic.dalto@tin.it (M.D.); am98@hotmail.it (A.M.); antonio.orlando1395@gmail.com (A.O.); 3 Department of Emergency Medicine, Santa Maria Delle Grazie Hospital, 80078 Pozzuoli, Italy; 4 Department of Translational Medical Sciences, University of Campania “Luigi Vanvitelli”, Monaldi Hospital, 80131 Naples, Italy; antoniopio.montella@unicampania.it (A.P.M.); luigi.pellegrinoo7@gmail.com (L.P.); giuseppe_boccaforno.563851@unifg.it (G.B.); marisa.defeo@unicampania.it (M.D.F.); 5 UOC Cardiac Surgery Vanvitelli, Monaldi Hospital, 80131 Naples, Italy; veronica.doria18@gmail.com; 6 Department of Mental and Physical Health and Preventive Medicine, University of Campania “Luigi Vanvitelli”, 80131 Naples, Italy; renato.franco@unicampania.it (R.F.); andrea.ronchi@unicampania.it (A.R.); 7 UOC Clinical Biochemistry and Clinical Pathology, Monaldi Hospital, 80131 Naples, Italy; carmine.nicastro@ospedalideicolli.it

**Keywords:** microplastics, nanoplastics, cardiovascular disease, cardiac pathophysiology, inflammatory response, endothelial dysfunction, echocardiography

## Abstract

The global spread of micro- and nanoplastics (MNPs) has emerged as an environmental and medical concern, with growing evidence of their role in cardiovascular disease (CVD). These particles, originating from the degradation of larger plastics and consumer products, can be ingested or inhaled, cross biological barriers, and accumulate in human tissues, including blood, myocardium, and atherosclerotic plaques. Experimental and clinical studies suggest that MNPs contribute to CVD through multiple mechanisms: activation of systemic inflammation and inflammasomes, oxidative stress, endothelial dysfunction, prothrombotic activity, and direct myocardial injury, ultimately promoting fibrosis and impaired contractility. Epidemiological data further indicate that populations exposed to higher plastic pollution or with pre-existing cardiovascular risk factors may be particularly vulnerable. Taken together, these findings identify MNPs as a potential novel environmental cardiovascular risk factor. Advancing detection methods, mechanistic research, and public health strategies will be essential to mitigate their impact and reduce plastic-related cardiovascular burden.

## 1. Introduction

The global proliferation of micro- and nanoplastics (MNPs) in the environment has become a growing concern for public health. These small plastic particles, derived from the degradation of larger plastic materials, enter ecosystems and human bodies through air, water and food sources [1,2]. While their ecological impact has been widely investigated, MNPs are increasingly implicated in human health, with emerging links to cardiovascular disease (CVD) and impaired myocardial function [3]. Accordingly, it is crucial to understand how exposure to MNPs contributes to the development and progression of CVD, focusing on mechanisms involving systemic inflammation, oxidative stress, endothelial dysfunction and direct and indirect heart damage [4].

Microplastics are plastic particles typically less than 5 mm in diameter; nanoplastics are even smaller (and potentially more dangerous for this reason) and are generally defined as particles smaller than 100 nanometers [5]. Beyond the contamination of food and drinking water, both types of particles arise from fragmentation of larger plastics in the environment, microbeads in personal care products, and synthetic fibers released during laundering [6,7]. Due to their minute size and high mobility, MNPs can be ingested or inhaled, and may traverse biological barriers, with evidence of deposition in multiple tissues, including the heart and vascular system [8].

Ingestion, inhalation, and dermal contact are the main pathways of human exposure to MNPs, with ingestion representing the predominant route. Within the gastrointestinal tract, these particles can interact with biomolecules such as proteins and lipids, influencing their absorption and bioavailability. MNPs have entered the food chain and been detected in various commonly consumed products, including honey, beer, salt, sugar, seafood, and bottled water. Seafood is of particular concern due to bioaccumulation along trophic levels. In addition, food packaging materials—especially those made of polymers such as PE, PP, PET, and PS—represent another relevant source of human exposure [9].

Although a large body of existing research has primarily focused on polystyrene (PS) nanoparticles, it is equally important to investigate other common polymers such as polypropylene (PP), polyethylene (PE), and polyethylene terephthalate (PET), given their extensive presence in the environment. The challenge in understanding their potential health effects lies in the diversity of their physical and chemical characteristics, which render them complex stressors with broad implications for and human health.

Recent biomedical studies have reported MNPs in human blood, myocardium, vascular tissues and atherosclerotic plaques, supporting a plausible link between internal contamination and CVD pathogenesis [10]. The detection of these particles in biological tissues is largely facilitated by advanced imaging techniques, such as gas chromatography–mass spectrometry and electron microscopy, which provides the high-resolution capability necessary to visualize and analyze MNPs at the nanoscale. Scanning Electron Microscopy and Transmission Electron Microscopy are commonly employed to identify the morphology and composition of these particles, revealing their interaction with cells and tissues [11]. The ability of gas chromatography—mass spectrometry and of electron microscopy to precisely detect MNPs underscores its importance in ongoing research, aimed at understanding the potential impacts of plastic particles on human health [12].

The main purpose of this review is to summarize the existing data and a perspective on their possible implications, examining the evolving medical knowledge on MNPs-mediated cardiovascular damage, with particular focus on cardiac ultrasound findings, tissue inflammation and systemic inflammatory responses.

## 2. The Socioeconomic Burden

Global plastic production reached 400.3 million metric tons in 2022, and projections estimate that approximately 13.2 billion tons of plastic waste will have accumulated in ecosystems by 2050. The widespread environmental dissemination of MNPs has shifted from being primarily an ecological concern to representing an urgent medical challenge [13]. Emerging epidemiological evidence suggests a potential association between environmental plastic exposure and adverse cardiovascular outcomes [14].

Populations living in regions with elevated levels of plastic pollution exhibit higher incidences of CVD; however, more research is required to establish causality and to delineate the magnitude of risk attributable to MNPs. Human studies have already detected MNPs in blood samples, suggesting that chronic exposure may facilitate their accumulation within the cardiovascular system over time [15].

Certain populations may be particularly vulnerable to the cardiovascular effects of MNPs due to a combination of environmental, genetic and lifestyle factors. Individuals with preexisting cardiovascular conditions, such as hypertension, hyperlipidemia, or diabetes, may experience amplified effects from plastic particle exposure. Similarly, those living in regions with high air and water pollution are likely to encounter greater MNPs loads, increasing CVD risk [16]. Socioeconomically disadvantaged communities, which comprise a substantial share of the global population, have shown heightened susceptibility to the adverse health impacts of MNPs contamination [17]. Older adults may be at greater risk owing to age-related declines in cardiovascular and immune function and, potentially, cumulative lifetime exposure to MNPs. Finally, infants and children—with developing organs and immature immune systems—may also be particularly vulnerable, although further research is needed to define the magnitude of these risks [18].

The potential health risks associated with MNPs, particularly in relation to CVD, underscore the need for more stringent regulation and systematic monitoring of plastic pollution. Current regulatory frameworks have largely targeted macroplastics and visible waste streams. There is growing recognition that MNPs in air, water, food, and consumer products must also be addressed through harmonized detection methods, exposure standards, and surveillance systems. Reflecting its importance for human and planetary health and building on the Minderoo–Monaco Commission’s statements [19], The Lancet journal has launched the “Lancet Countdown on Health and Plastics” [20]. This indicator-based initiative tracks exposures, policy responses, and health outcomes, and issues regular public reports on progress in reducing MNPs-related risks.

## 3. The Mechanisms of Cardiovascular Impact

Human exposure to MNPs occurs mainly through ingestion of contaminated food and water, and inhalation of airborne particles, and to a lesser extent by dermal contact [21]. Owing to their small size, MNPs can cross biological epithelial barriers, enter systemic circulation, and distribute to tissues. Their presence in human blood was first confirmed in 2019, and subsequent studies have reported mean concentrations of approximately 1.6 μg/mL [22]. With limited human enzymatic pathways for polymer degradation, MNPs are biopersistent and prone to bioaccumulate [23]. Emerging evidence points to significant cardiovascular involvement [24]: MNPs have been detected in blood, atherosclerotic plaques, heart valves, and myocardial tissue, with their presence correlating with adverse cardiovascular outcomes. The cardiovascular system appears particularly vulnerable to bioaccumulation. MNPs have been detected in human pericardial tissue, myocardium and venous blood from patients undergoing cardiac surgery, confirming translocation beyond the vasculature into cardiac structures [25]. Animal models demonstrate MNPs crossing the placental barrier and accumulating in fetal cardiac tissue, suggesting potential developmental cardiovascular toxicity. Furthermore, clinical studies increasingly associate MNPs exposure with measurable cardiovascular pathology and adverse events. MNPs concentrations were highest in patients with acute myocardial infarction compared to unstable angina and correlated positively with coronary artery disease complexity assessed by the SYNTAX score [26]. This suggests a potential dose–response relationship between MNPs burden and ischemic severity. Pyrolysis–gas chromatography/mass spectrometry (Py–GC/MS) analyses reveal polyethylene (PE) as the predominant polymer, followed by polyvinyl chloride (PVC), polystyrene (PS), and polypropylene (PP). In addition, landmark analyses of carotid endarterectomy specimens identified MNPs, PE, and PVC within plaques. Electron microscopy showed irregular, jagged-edged particles embedded in macrophages and necrotic debris. In a prospective cohort of 257 patients with carotid stenosis followed for 34 months [27], the presence of MNPs in excised plaques independently predicted major adverse cardiovascular events (myocardial infarct, stroke, cardiovascular death) with a hazard ratio of 4.53 (95% CI 2.00–10.27). MNP-positive plaques also displayed features of vulnerability, including heightened inflammatory infiltrates.

**Figure 1:**
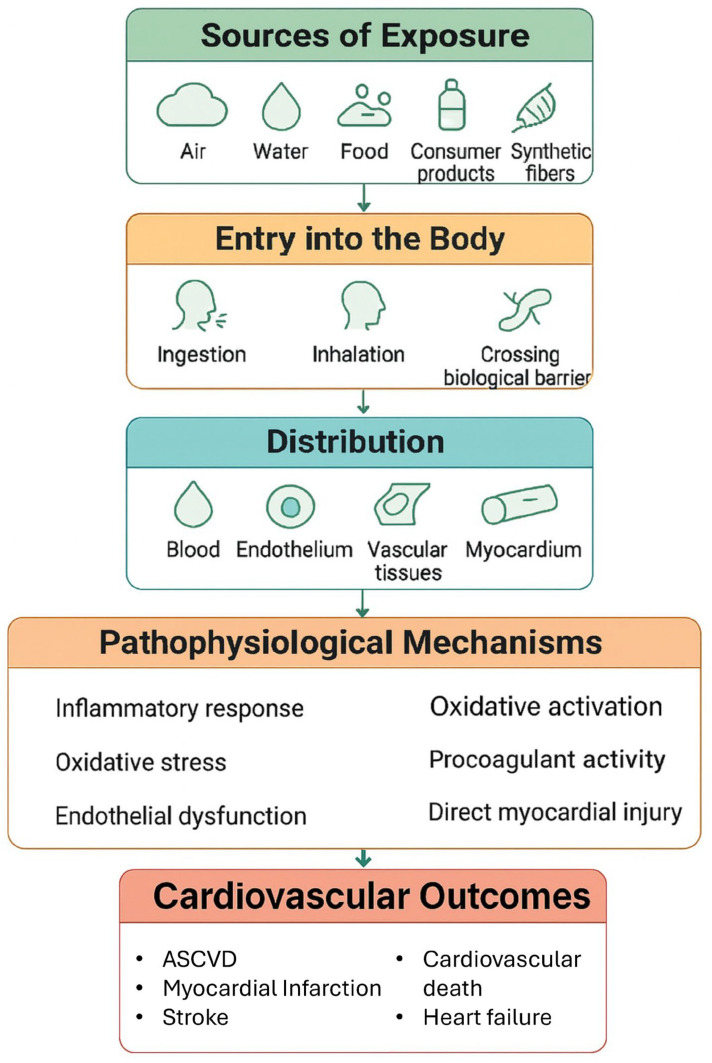
Main pathways Linking MNPs Exposure to CVD. ASCVD: Atherosclerotic Cardiovascular Disease.

From a pathophysiological standpoint, the cardiovascular system response to MPs is complex and involves multiple pathways, such as inflammation, oxidative stress, endothelial dysfunction, procoagulant activity, and direct myocardial injury Figure 1 [28].

### 3.1. Inflammatory Response

Once internalized, MNPs can trigger an immune reaction, with macrophages attempting to engulf and degrade these foreign particles. This process promotes the release of proinflammatory cytokines and chemokines, contributing to a persistent state of low-grade inflammation, which is a well-established risk factor for CVD [29,30]. MNPs, particularly environmental particles rich in polyethylene terephthalate (PET), are potent activators of the NLRP3 inflammasome in macrophages and endothelial cells. Activation of this multiprotein complex drives the cleavage and secretion of key proinflammatory cytokines, primarily interleukin-1β (IL-1β) and IL-18 [31]. In vitro studies further demonstrate that exposure to MNPs significantly increases the release of IL-1β, IL-6, IL-12p70, and tumor necrosis factor-alpha (TNF-α) from human immune and vascular cells. IL-6 serves as a central regulator of CRP production, linking local vascular inflammation to systemic acute phase responses. Elevated cytokine levels are closely associated with plaque instability, endothelial dysfunction and adverse cardiac remodeling [32,33]. Moreover, flow cytometry analyses in acute coronary syndrome patients revealed that higher circulating MNPs levels correlate with increased counts of B lymphocytes and natural killer (NK) cells, alongside altered monocyte profiles. MNPs can also be internalized by macrophages, promoting their differentiation into proinflammatory foam cells upon exposure to oxidized LDL (ox-LDL),a hallmark of atherosclerotic lesions. Chronic exposure to MNPs may further drive macrophage polarization toward a proinflammatory M1 phenotype within vascular tissues, amplifying vascular inflammation and disease progression [34].

### 3.2. Oxidative Stress

Direct interaction between MNPs, particularly nanoplastics, and vascular or cardiac cells leads to excessive generation of reactive oxygen species (ROS) [35]. This overwhelms endogenous antioxidant defenses (e.g., glutathione, superoxide dismutase), resulting in oxidative damage to lipids (such as LDL oxidation forming proatherogenic ox-LDL), proteins, and DNA [36]. The ensuing oxidative stress impairs endothelial function through adhesion to endothelial cells, thus disrupting their physiological function and activating both inflammatory pathways and oxidative stress responses [37].

### 3.3. Endothelial Dysfunction

The vascular endothelium represents a primary target of MNP-induced injury. Experimental evidence demonstrates that exposure disrupts endothelial cell function, increasing vascular permeability, and reducing nitric oxide (NO) production, which in turn impairs vasodilation [38]. MNPs exposure also promotes endothelial activation, characterized by upregulated expression of adhesion molecules (VCAM-1, ICAM-1, E-selectin). This promotes leukocyte (monocyte, neutrophil) adhesion and transmigration into the subendothelial space, initiating and sustaining vascular inflammation. MNPs also impair endothelial nitric oxide (eNO) bioavailability, which is crucial for vasodilation and vascular homeostasis, ultimately contributing to vasoconstriction and hypertension [39].

### 3.4. Procoagulant Activity

Beyond their contribution to endothelial dysfunction, MNPs have been implicated in enhanced thrombotic activity and blood clot formation [40]. They can directly activate platelets, leading to a heightened risk of clot development [41]. In individuals with atherosclerosis or other vascular conditions, this prothrombotic effect could significantly increase the risk of acute cardiovascular events. The procoagulant potential of MNPs is thought to arise from their capacity to induce oxidative stress and inflammation, both of which are well-established activators of platelet function and the coagulation cascade. Moreover, the physical presence of MNPs within the vascular lumen may act as a nidus for clot formation, providing surfaces that facilitate platelet adhesion, aggregation, and fibrin deposition [42].

### 3.5. Direct Myocardial Injury

In addition to vascular effects, MNPs may exert direct cardiotoxicity. Advanced imaging techniques such as electron microscopy have been revealed MNPs accumulation within myocardial tissue, both intracellularly and in the extracellular matrix. This localization correlates with structural and functional alterations, including myocardial fibrosis. Fibrosis, defined by the excessive extracellular matrix deposition, compromises myocardial compliance and contractility, leading to impaired relaxation, reduced pumping efficiency, and increased arrhythmogenic potential [43]. Animal models demonstrate that polystyrene exposure promotes myocardial fibrosis, via multiple pathways, including TGF-β1/Smad and Wnt/β-catenin signaling, as well as ROS-mediated fibroblast activation, ultimately driving excessive collagen deposition [44]. Chronic exposure to MNPs, particularly polystyrene, has also been associated with increased adiposity, weight gain, and altered metabolic profiles in animal models, promoting a cardiometabolic phenotype that synergistically increases cardiovasculsar risk [45]. At the cellular level, sustained MNP exposure induces stress-related cellular senescence in endothelial and vascular smooth muscle cells. Senescent cells acquire a senescence-associated secretory phenotype, characterized by the release of proinflammatory cytokines, chemokines, and proteolytic enziymes tissue injury and perpetuate vascular inflammation. Furthermore, MNPs can trigger apoptosis or necrosis in cardiovascular cells, aggravating tissue damage and amplifying inflammatory responses (Table 1).

While no study has directly linked MNP exposure to pulmonary hypertension (PH), many of the identified mechanisms (inflammation, oxidative stress, endothelial dysfunction, procoagulant activity) are well-recognized drivers of PH pathogenesis. Otherwise, the vascular injury evidence (including increased arterial stiffness and endothelial cell dysfunction) suggests biologically plausible pathways that MNPs could contribute indirectly to development or progression of PH.

Table 2 summarizes the main reports, projects and studies that have been published in this area.

## 4. Current Research and Future Perspectives

Preclinical studies have provided consistent evidence that exposure to MNPs induces measurable alterations in cardiac function as assessed by echocardiography. In murine models exposed to polystyrene nanoplastics via inhalation, investigators reported a dose- and time-dependent decline in left ventricular systolic performance, with significant reductions in ejection fraction (EF) and fractional shortening (FS) after four to twelve weeks of exposure [46]. These functional impairments were accompanied by structural remodeling, including dilation of the left ventricular cavity (increased left ventricular diastolic and systolic diameter) and wall thinning, consistent with progressive contractile dysfunction. Additional preclinical data demonstrated that MNPs accumulation within myocardial tissue is associated with bradycardia, reduced stroke volume, and concentric remodeling, reinforcing the link between plastic particle exposure and impaired myocardial performance [46]. Collectively, echocardiographic findings in animal models highlight the cardiotoxic potential of MNPs, suggesting early subclinical functional alterations that may precede overt histopathological damage.

MNPs translocate into the circulatory system, accumulating in cardiac tissue and inducing multifaceted damage. Heart ultrasound, especially speckle-tracking (STE), myocardial work and three-dimensional (3D) techniques, could theoretically warrant a sharper assessment of heart functional effects of MNPs contamination.

More in detail, myocardial work, mechanical dispersion and Global Longitudinal Strain (GLS) are well-known sensitive markers of systolic function effectiveness and of atrioventricular contractile longitudinal function [47]. In routine clinical care, resting LVEF calculated by echocardiography is employed to investigate reduction in LVEF as a sign of explicit left ventricular dysfunction. Therefore, to prevent delay in management, there is a need for a reliable and sensitive marker for early recognition of cardiac damage. The myocardial function can vary significantly without changes in LVEF. Hence, 2D speckle tracking echocardiography (2D STE) has been used as a favorable tool to assess cardiac damage before left ventricular function alteration. Likewise, 3D-volumetric and strain analysis seem to be among the more promising fields of 3D-echocardiography for this purpose.

Even though several observations appear to support the potential interest in exploring the role of cardiac and vascular ultrasound in human MNP contamination, it must be clearly emphasized that any discussion of this topic remains largely speculative. Indeed, only limited and indirect data are currently available in humans, with the vast majority of evidence deriving from preclinical studies.

On the basis of these considerations, a prospective observational, double-blind, multicenter study is currently being conducted in the human heart to clarify the consequences of myocardial and blood contamination by MNPs. The primary endpoint is to assess the systemic inflammatory response associated with cardiovascular MNPs contamination. The secondary endpoint is to explore the functional impact of MNPs by means of comprehensive echocardiographic evaluation, including conventional parameters, speckle-tracking analysis, and three-dimensional echocardiography, in order to identify potential imaging signatures of MNPs exposure. The sample size was calculated to include 86 patients, following established methodological standards. Peripheral venous blood samples and myocardial tissue from anatomically relevant cardiac regions will be collected during cardiac surgery, stored according to standard histopathological protocols, and analyzed using gas chromatography–mass spectrometry and high-resolution electron microscopy. Structural findings obtained from histological and ultrastructural analyses will then be correlated with echocardiographic alterations observed in pathological hearts, with the aim of linking MNPs burden to in vivo functional impairment. In addition, each patient will undergo a thorough assessment of cardiovascular risk factors using validated scales, together with a complete biochemical profile (electrolytes, inflammatory markers, liver, renal and thyroid function, lipid panel, glucose metabolism, BNP/NT-proBNP). The trial has been approved by the local ethics committee and is currently in the enrollment phase.

The final results of this ongoing investigation are expected in the coming months and are anticipated to provide novel insights into the pathophysiological interplay between MNPs contamination, cardiac structure, and function.

## 5. Conclusions

The interplay between MNPs and CVD is increasingly recognized as a critical issue for human health. Accumulating evidence indicates that MNPs—particularly nanoplastics and polymers such as PET and PVC—drive systemic inflammation and vascular injury through oxidative stress, inflammasome activation, cytokine release, endothelial dysfunction, immune dysregulation, and cellular senescence or death. These mechanisms create a proinflammatory, prothrombotic, and profibrotic milieu that may contribute to atherosclerosis, acute coronary syndromes, and myocardial injury. Despite persisting knowledge gaps, converging clinical and experimental findings strongly suggest that MNPs exposure represents a potential novel environmental cardiovascular risk factor. Addressing this challenge requires a multipronged approach: advancing methods for sensitive detection of MNPs in biological matrices, conducting rigorous mechanistic and translational studies, and implementing public health strategies aimed at reducing plastic waste and human exposure. Ultimately, integrating scientific evidence with effective policy measures to limit plastic pollution will be essential to mitigate the cardiovascular risks posed by MNPs and to protect global health. From a preventive standpoint, mitigation strategies should aim at both reducing MNP release into the environment and limiting human exposure. At the individual level, behavioral measures such as minimizing the use of single-use plastics, improving water filtration, and favoring fresh rather than processed foods may decrease ingestion and inhalation of particles. At the population level, stricter regulatory policies, waste management improvements, and industrial innovation toward biodegradable materials represent crucial steps. The world-wide burden of MNPs pollution should be considered a main issue for the human health. Further clinical studies are urgently needed to improve specific knowledge, increasing in turn the awareness among physicians, ordinary people and political class about MNPs-related health risks.

## Figures and Tables

**Table 1:** Inflammatory mediators and pathways activated by MNPs in cardiovascular tissues.

Inflammatory Mechanism	Key Effectors	Cardiovascular Consequences
Oxidative Stress	ROS (Superoxide, H_2_O_2_), Oxidized LDL	Endothelial dysfunction, ox-LDL uptake → Foam cells, DNA damage → Senescence
Inflammasome Activation	NLRP3, Caspase-1, IL-1β, IL-18	Plaque inflammation and instability, Pyroptosis, Systemic inflammation
Cytokine/Chemokine Release	IL-6, TNF-α, IL-12p70, IL-8, MCP-1	Systemic acute phase response, Leukocyte recruitment, Endothelial activation
Immune Cell Activation	↑ Monocytes/Macrophages (M1), ↑ B-cells, ↑ NK cells	Foam cell formation, Chronic vascular inflammation, Antibody production, Cytotoxicity
Endothelial Dysfunction	↑ VCAM-1/ICAM-1/E-selectin, ↓ eNO	Leukocyte adhesion, Vasoconstriction, Hypertension, Thrombosis risk
Cellular Senescence/Death	SASP, Apoptosis, Necrosis (Microsoft Word, Version 16.89 (24090815), Licence: Microsoft 365 subscription)	Tissue remodeling, Plaque rupture, Myocardial Fibrosis

↑: increase; ↓: decrease.

**Table 2:** Summary of the main reports, projects, and studies published in the field of MNPs.

First Author	Study Type	Study Field	Main Findings	Limitations	Ref
Abbas G	review	human (both in vitro and in vivo)	focusing on relevance of MNP toxicity and policy implications	propositive only	[1]
Afzal Z	review	human (both in vitro and in vivo)	comprehensive overview of MNP-induced cardiovascular damage mechanisms	none	[3]
Zhang T	review	human (both in vitro and in vivo)	linking MNP-contamination mechanisms and public health strategies	very large area	[16]
Zhu X	review	animal and human (both in vitro and in vivo)	focusing the connection among MNP absorption and cardiovascular harm	limited data available	[18]
Landrigan PJ	scientific-based project	human (in vivo)	eye-opening recommendations against MNP toxicity	none	[19]
Landrigan PJ	scientific-based project	human (in vivo)	world-wide prospective monitoring on MNP threat	none	[20]
Yang Y	prospective	human (in vivo)	sharp detection of heart contamination by MNP	incomplete tissue samples collection	[21]
Marfella R	prospective	human (in vivo)	first demonstration of MNP effect on morbidity-mortality	one cardiovascular district investigated only	[27]
Bishop B	prospective	human (in vitro)	focusing the different effect of environmental and commercial PNMs on human cells inflammation an death	lack of data on the complex inflammatory cascade in vivo	[31]
Chowdhury SR	review	human (both in vitro and in vivo)	addressing the link between MNP exposure and functional heart parameters	speculative:, lack of available data	[24]
Mattioda V	prospective	animal and human (in vitro)	focusing MNP exposure, inflammation and cytotoxicity in the same model	in vitro data	[30]
KC PB	prospective	human (in vitro)	addressing the pathway leading from inflammation to cytotoxicity	in vitro data	[32]
Lomonaco T	prospective	human (in vitro)	focusing oxdative stress and inflammation in the same model	in vitro data	[38]

## Data Availability

No new data were created or analyzed in this study. Data sharing is not applicable to this article.

## Abbreviations

The following abbreviations are used in this manuscript:

CVD	Cardiovascular Disease
IL	Interleukin
LVEF	Left Ventricle Ejection fraction
MNPs	Microplastics and Nanoplastics
NO	Nitric Oxyde
PE	Polyethylene
PH	Pulmonary Hypertension
PVC	Polyvinyl Chloride
ROS	Reactive Oxygen Species
STE	Speckle-tracking Echocardiography
